# X-ray topography of subsurface crystal layers^1^

**DOI:** 10.1107/S1600576717007208

**Published:** 2017-05-30

**Authors:** Zbigniew Swiatek, Igor Fodchuk, Ruslan Zaplitnyy

**Affiliations:** a Institute of Metallurgy and Materials Science, 25 Reymonta Street, Krakow, 30-059, Poland; b Solid State Physics Department, Yuriy Fedkovych Chernivtsi National University, Kotsyubynskiy Street 2, Chernivtsi, 58018, Ukraine

**Keywords:** X-ray diffraction, X-ray topography, structural diagnostics, ion implantation, Berg–Barret method

## Abstract

New capabilities of the Berg–Barrett topographic method are demonstrated using a skew-asymmetric X-ray diffraction scheme for investigating structural changes near the surface of semiconductor materials. Specifying the X-ray extinction depth, the details of defects and strains are revealed with high resolution. Consequently, analysis of structural distortion of layers near the surface after various types of surface processing becomes more complete.

## Abstract 2

The practical application of the modified Berg–Barrett topographic method in a skew asymmetric scheme of X-ray diffraction is presented. The method is used for the study of the defect structure of CdTe crystals and Cd_1−*x*_Hg_*x*_Te/CdTe epitaxial layers after the influence of different external factors (ion implantation and etching).

## 1. Introduction   

The fundamentals of X-ray diffraction topography were formulated by Berg (1931 ▸) and supplemented in the work of Barrett (Barrett, 1945 ▸; Ramachandran, 1944 ▸). Since then, a lot of X-ray optical schemes have been applied in various methods of X-ray topography and diffractometry (Authier, 2001 ▸; Bedyńska, 1973 ▸; Tanner, 1976 ▸; Bowen & Tanner, 1998 ▸; Holy *et al.*, 1999 ▸).

The improvement of the known methods of studying the structure of materials and the creation of new methods is still relevant today. This is especially true for X-ray diffractometry and topography. In recent years, these methods have changed significantly in parallel with advances in the development of the dynamical theory of X-ray diffraction by crystals with defects and computer simulation (Härtwig, 2001 ▸; Holy *et al.*, 1999 ▸). High-resolution sectional and projection double-crystal topography (Authier, 2001 ▸; Bowen & Tanner, 1998 ▸; Indenbom & Kaganer, 1985 ▸; Novikov *et al.*, 1995 ▸; Petroff, 1984 ▸) is sufficiently effective in the investigation of various heterostructures and multilayer systems. It presents an opportunity to study the features and mechanisms of formation of diffraction contrast of one- and two-dimensional defects on the surface and in the volume of crystals with higher resolution.

White-beam synchrotron topography (Bowen & Tanner, 1998 ▸) is highly effective for the study of defects in materials. A relatively simple implementation of the experimental scheme can easily be adapted for real-time studies of the dynamical effects on materials in *in situ* control mode. Such a topography scheme, because it involves the analysis of multiple reflections, allows us to obtain maps of complete long-range elastic deformations from various defects. In this case, a double-crystal scheme of topography with the use of several reflections enables the determination of the dilatations and angular misorientations (Riglet *et al.*, 1980 ▸). At the same time, a direct method for plotting misorientation and dilatation maps requires an installation of the crystal analyzer after the sample (Kitano *et al.*, 1991 ▸). The sequential movement of the analyzer makes it possible to build complete dilatation maps by the method of multiple exposures.

To characterize the three-dimensional structure of defects in a diamond single crystal, a combination of diffraction topography and microtomography is quite effective (Ludwig *et al.*, 2001 ▸). The use of the rocking curve method is also valid to study rotational and dilatation components of the lattice strain in silicon crystals by analyzing the dependences of the integrated intensity distribution, the half-width of the reflection curve and the peak intensity on the angular deviation from the Bragg angle (Tsoutsouva *et al.*, 2015 ▸). In addition, various modifications of the topographic Berg–Barrett method are still quite effectively used today to estimate the quality of the structure of semiconductor materials (Black & Long, 2004 ▸; Lübbert *et al.*, 2000 ▸; Lübbert & Baumbach, 2007 ▸; Swiatek & Fodchuk, 2016 ▸).

In this paper new possibilities of the X-ray topography method in the skew-asymmetric Bragg diffraction scheme (which are achieved by specifying the X-ray extinction depth) are shown for studying the morphology of the surface of semiconductor materials and structural changes after various types of surface processing.

## 2. Grazing-incidence skew-asymmetric Bragg topography: experimental setup, geometrical conditions and principal relationships   

**Figure 1:**
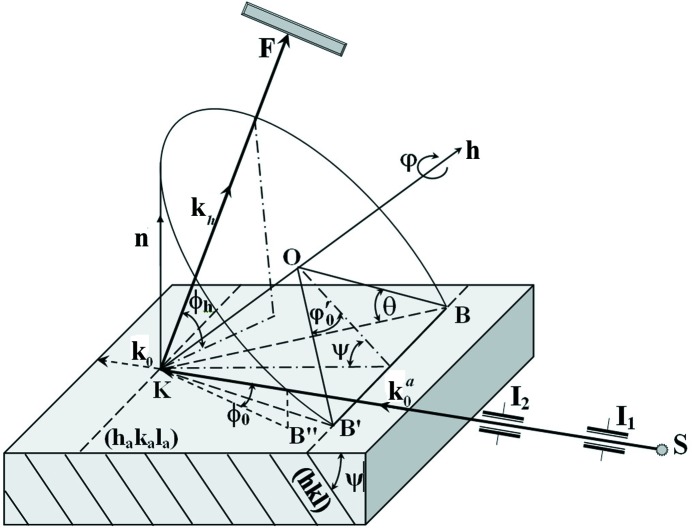
Experimental scheme of skew-asymmetric geometry of topography. S is the X-ray source, 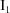 and 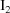 are the collimation slits, F is the film, and K is the crystal.

There are a large number of different X-ray techniques that allow us to study thin subsurface layers with a thickness of about 0.1 µm (Afanas’ev & Melkonyan, 1983 ▸; Afanas’ev *et al.*, 1989 ▸; Authier, 2001 ▸; Bowen & Tanner, 1998 ▸; Imamov *et al.*, 1989 ▸; Novikov *et al.*, 1995 ▸). It is also highly efficient to use asymmetric reflections followed by rotation of the sample around the diffraction vector (Grigoriev *et al.*, 2016 ▸). But no less effective is the diffraction scheme for which the angle ψ between the reflecting plane and the crystal surface is slightly larger than the value of the Bragg angle θ (Fodchuk & Kshevetsky, 1992 ▸; Fodchuk *et al.*, 1995*a* ▸, 2009 ▸; Swiatek & Fodchuk, 2016 ▸):
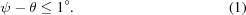
The position of the crystal when both the normal 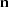 to the crystal surface and the diffraction vector 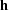 lie in the same plane of diffraction is typical when the condition of extremely asymmetric Laue diffraction is realized. During rotation of the crystal by φ around 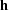, the vector 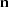 circumscribes a cone (Fig. 1 ▸). In this way the Bragg condition for the incident beam can be achieved; so, 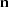 does not lie in the plane of diffraction and various angles between the incident beam and the surface can be realized (Fig. 1 ▸ and 2 ▸). In previous work (Swiatek & Fodchuk, 2016 ▸) it has been noted that this technique of topography opens up new possibilities for layer-by-layer visualization of structural changes in the near-surface layers of a crystal and can also be used in a double-crystal arrangement for determining the strain values in near-surface layers (Fodchuk & Kshevetsky, 1992 ▸; Fodchuk *et al.*, 1995*b* ▸).

The directional cosines for the angles of incidence (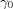) and reflection (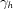) are determined as functions of the azimuthal scan angle φ (Fodchuk *et al.*, 1995*a* ▸). The asymmetric diffraction in the non-skew condition, *i.e.* when all vectors 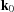, 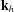, 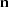 and 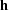 lie in the diffraction plane, is a reference point for the angle φ:
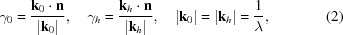
where λ is the X-ray wavelength.

**Figure 2:**
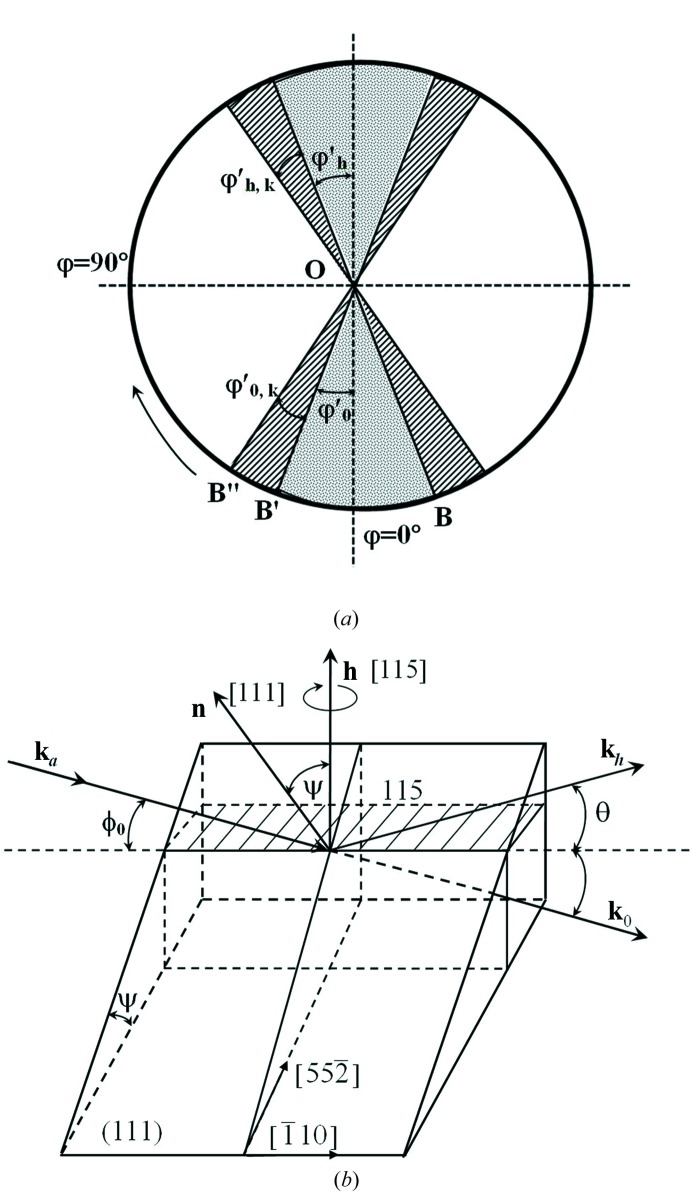
(*a*) Diagram of the transition from the Laue condition to the Bragg condition during rotation of the crystal by φ around 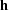: 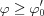, 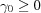; 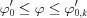 corresponds to the simultaneous total external reflection of the incident beam 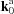 from the surface and diffraction from the (*hkl*) plane. (*b*) Skew-symmetric case of X-ray diffraction (this example shows a CdTe crystal, Cu 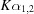 radiation): 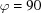
°, 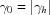; surface is (111), diffracting plane is (115).

In this case the directional cosines for the angles of incidence (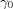) and reflection (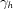) are determined as functions of angles θ, ψ and φ:
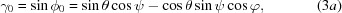


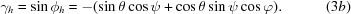
When 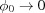 we can determine the boundary value 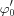 of the angle φ from the ratio (3*a*) (Fig. 2 ▸). This angle corresponds to the transition from the Laue condition to the Bragg condition:
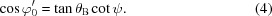
When 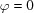
° the case of asymmetric Laue diffraction is realized (Fig. 2 ▸):
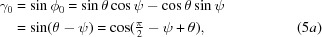


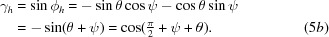
It is easy to determine from relation (2) that the transition from the Laue condition to the Bragg condition (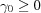) will occur at angles greater than 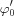 (Fig. 2 ▸):
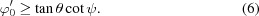
When 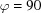
°, the case of skew-symmetric Bragg diffraction occurs (Fig. 2 ▸) and
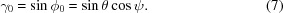
Moreover, at angles ranging from 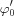 to 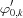 the beam reflected by total external reflection can appear in addition to the diffracted beam (Brümmer *et al.*, 1976 ▸).

To implement the geometrical condition of skew-asymmetric diffraction we should choose the diffraction planes (*hkl*) for which the difference between the values of θ and ψ is negligible. The diffraction planes satisfying this condition for the standard X-ray setups and wavelengths of characteristic X-ray radiation are given in Table 1 ▸.

The range of azimuthal angles φ for which 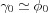 (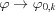) is within several degrees. This allows us to use a simple technical adjustment to change the incident angles (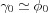) gradually and accurately. Of greatest interest for topography are the angle values near the angle of total external reflection, 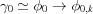 (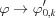), when the incident beam is almost grazing over the surface (Figs. 1 ▸ and 2 ▸). Then, we can achieve the following:

(*a*) An increase of the extent of the wavefront for the diffracted beam in comparison with the incident beam (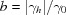) gives the possibility to obtain topograms from thin near-surface layers of comparatively large area in a single crystal (Swiatek & Fodchuk, 2016 ▸). It is important that the angle of diffraction (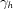) is rather wide (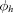 ≥ 80°). Consequently, we can obtain images virtually without distortion if the photographic material or detector is close to the sample.

(*b*) A decrease of 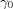 leads to a decrease of the penetration depth of X-rays into the near-surface layers of the crystal, which is determined by the extinction length (Hartwig, 1978 ▸; Rustichelli, 1975 ▸)
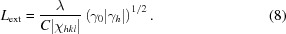
Here, λ is the wavelength; 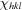 is the Fourier component of the crystal polarizability, calculated according to the algorithm proposed by Stepanov (2004 ▸); and 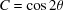 or 1 for π and σ polarization, respectively. For an unpolarized wave (the case of an X-ray tube) 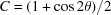.

## 3. Experimental conditions   

The effectiveness of this method depends not only on the geometry and features of X-ray scattering by crystals but also on the experimental conditions: the spectral composition of the radiation, the size and shape of the source S, the distances from the source to the crystal SK and from the crystal to the photographic film (or detector) KF, and the resolution of the photographic material.

In our case, the standard X-ray topography setup was used (Fig. 1 ▸), the area of the sharp focus source was *S* = 250–300 µm^2^, SK = 0.6 m, KF = 0.01 m, the resolution of the photoemulsion was 300 dashes per millimetre and the emulsion thickness was 25 µm.

To reduce the angular divergence of the beam in the plane of incidence (in the horizontal plane), two systems of collimating slits 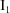 and 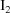 (width of 2 mm and height of 50 mm) were used, both in front of the source and in front of the crystal. The total spatial resolution of the image (taking into account all instrumental errors) of the photographic material was 5–7 µm in the direction normal to the crystal surface.

In some cases work was carried out in a double-crystal arrangement in non-dispersive mode in order to obtain and analyze rocking curves. Crystals of Si with asymmetric 220 and InSb with symmetric 333 reflections were used as monochromators.

This geometry of reflection topography makes it possible to selectively visualize structural changes in the near-surface layers of crystalline compounds after different external influences with a sufficiently small step (0.05–0.1 µm) of depth penetration.

## 4. Results   

The potential of X-ray topography to record defects in the crystal lattice is associated with different types of contrast: adsorption, orientation and extinction. These contrast types can be separated from each other in most cases (Authier, 2001 ▸; Petroff, 1984 ▸).

The absorption contrast is due to differences between the absorption coefficients in different areas of the sample.

The orientational contrast is determined by the difference between reflected intensities from neighboring local ranges with different orientation of the crystal lattice. To describe the orientational contrast a very good approximation applicable in a very wide range has been proposed by Bonse (1962 ▸):
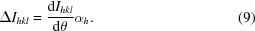
Here 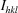 is the intensity of the diffracted beam and 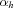 is the lattice misorientation. An increase of the sensitivity of topography with orientational contrast to defects can be achieved by improving the collimation of the characteristic X-ray beam or its monochromatization (Tanner, 1976 ▸).

The formation of the extinction contrast is associated with a different degree of attenuation of the incident and reflected beams due to a change in their interaction in the perfect and deformed regions of the crystal. In this case, the region with the strain gradient can be considered as a set of mosaic elements, *i.e.* as a mosaic crystal in which the dynamic interaction of waves is reduced, and hence the intensity of the beam reflected by this region differs from the intensity reflected by the surrounding (perfect) crystal (Kuriyama & Long, 1984 ▸).

It should be noted that in topographic studies the absorption coefficient of the radiation is of great importance, since it determines the depth of X-ray penetration and the features of image formation on topograms. In our case, soft X-ray radiation is more effective (Table 1 ▸).

**Figure 3:**
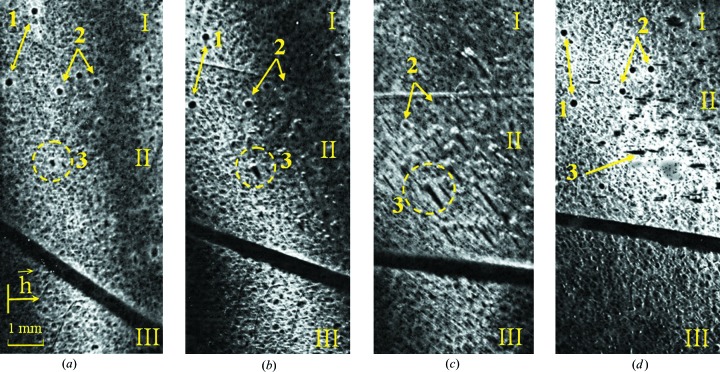
Mercury ion etching of variable-gap Cd_1−*x*_Hg*_x_*Te/CdTe structure (*x* = 0.19). The accelerating voltage of the Hg ions is (I) *E* = 1200 V, (II) *E* = 2400 V and (III) *E* = 600 V. Crystal surface is (111), reflection is 511, Cu 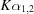. (*a*) 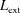 = 2.61 µm (φ = 35°); (*b*) 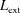 = 2.17 µm (φ = 25°); (*c*) 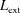 = 1.11 µm (φ = 18°); (*d*) 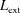 = 1.42 µm (φ = 20°). Numbers 1 and 2 indicate cavities of hexagonal shape, and 3 indicates hill-like inclusions of another phase above the surface.

**Figure 4:**
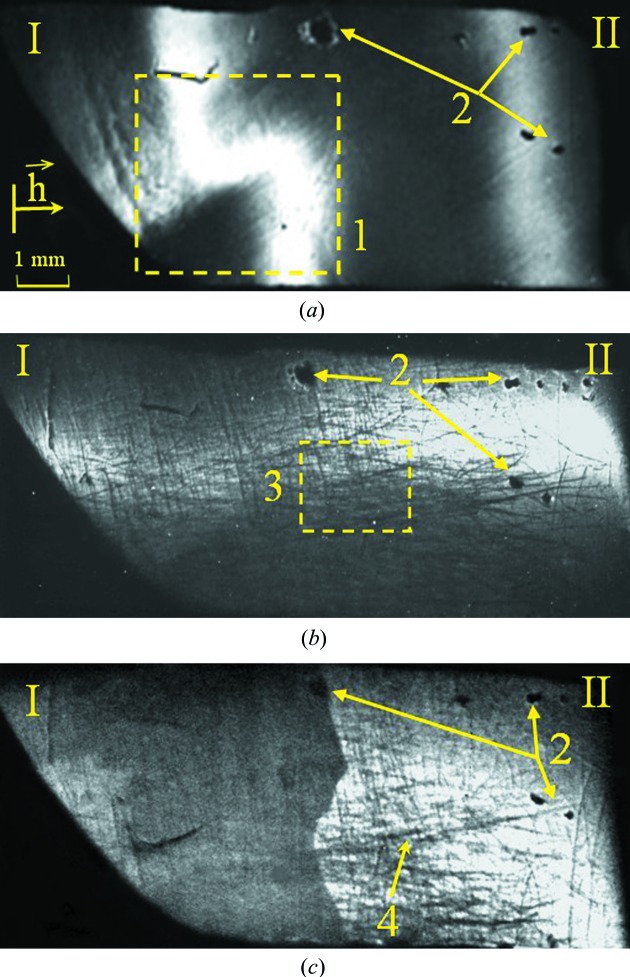
CdTe(111). Region I contains implanted As ions (*E* = 100 keV, *D* = 10^15^ cm^–2^). Topograms were obtained with Cu 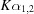 radiation. (*a*) Asymmetric 440 reflection, 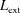 = 3.68 µm; (*b*) skew-asymmetric 511 reflection, 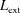 = 2.95 µm (φ = 30°); (*c*) skew-asymmetric 511 reflection, 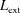 = 1.65 µm (φ = 20°). Numbers 1 indicates structure fragmentation, 2 cavities, 3 micro-scratches and 4 hill-like inclusions.

**Figure 5:**
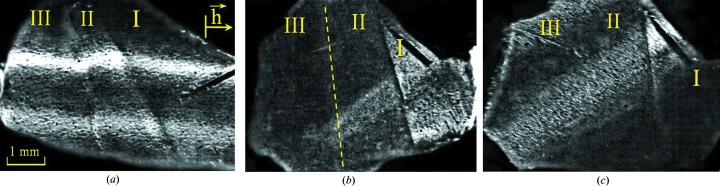
Topograms of Cd_1−*x*_Hg*_x_*Te/CdTe (111) epitaxial layers, obtained with the skew-asymmetric 115 reflection of Cu 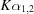. Region I is not implanted, region II is implanted with dose *D*_1_, and region III is implanted with dose *D*_1_ + *D*_2_. (*a*) 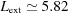
 µm (φ = 90°); (*b*) 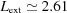
 µm (φ = 30°); (*c*) 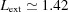
 µm (φ = 20°).

Figs. 3–5 show examples of the application of the skew-asymmetric scheme of reflection topography for different crystals of CdTe and its compounds.

### 4.1. Mercury ion etching of variable-gap structure   

The method of plasma-chemical etching is based on the chemical reactions between atoms or molecules of materials and ions of chemically active gases or vapor (Savitsky *et al.*, 1998 ▸). This method has a high degree of selectivity of etching materials and is not inferior to liquid-chemical etching (Tennant *et al.*, 1992 ▸). After plasma-chemical etching, as well as after ion etching, additional cleaning, washing and drying of the samples are not required.

Fig. 3 ▸ shows the results of topographic studies of the effect of mercury ion etching from a high-frequency mercury glow discharge on the morphology of the (111) Cd_1−*x*_Hg*_x_*Te/CdTe surface. The following values of the accelerating voltage of Hg ions were used: I – 1200 V; II – 2400 V; III – 600 V.

Two systems of equivalent ‘skew-symmetric’ planes (511) and (151) were used for topographic studies, for which 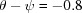
° (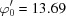
°).

According to relation (8), at the transition from a skew-symmetric to an extremely skew-asymmetric scheme, 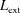 is reduced several times from 6.74 to 1.12 µm. In this case, the Cu 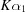 and Cu 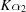 reflections are broadened, and the values of their maximum intensities are decreased. The surface morphology in irradiated regions is different. Mechanical damage (micro-scratches) is observed in region III; at the same time it is absent in regions I and II.

The difference in the reflectivity of the irradiated regions increases in the topograms with decreasing 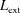 (Fig. 3 ▸ *b*). Cu 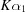 and Cu 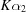 reflections suffer minor displacements and blurring on boundaries between the regions. In region II, where the ion energy is the largest, ion etching has a pronounced selectivity. This is evident in the significant rise of hill-like inclusions of another phase (indicated by number 3) above the etched matrix and the appearance of cavities of hexagonal shape (indicated by numbers 1 and 2) with a light edge. This is especially well illustrated in Fig. 3 ▸(*c*) at 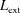 = 1.1 µm. In the same part of the crystal, the presence of structural micro-misorientation between local regions is indicated by the changing angular distances between the Cu 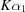 and Cu 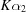 reflections and their bending.

The height *s* of a single hill can be estimated knowing the length of its shadow *l* on the surface by the ratio
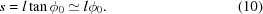
In our case, the height range of the hills changes from 0.1 to 12 µm. The minimum hill size detected by this technique depends on the resolution of the photographic material or CCD camera. If, for example, the shadow length is *l* = 1 mm, then in the range near to angles of total eternal reflection (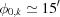) 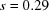
 µm.

Analysis of the rocking curve characteristics (half-widths, maximum values and integral intensity) allows evaluation of the average strain values in the layer, the thickness of which corresponds to the extinction length (Fodchuk *et al.*, 2007 ▸). Starting with 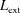 ≃ 2.6 µm (Fig. 3 ▸), the values of the half-widths *W* of the rocking curves are significantly increased in regions I and II in comparison with region III. Values of strains were averaged over the layer thickness. The ratio between the strains in the three regions is 1:2.3:1.5 at 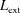 ≃ 1.4 µm and 1:4:1.8 at 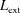 = 1.1 µm. In general, this corresponds to the values of the accelerating voltage for the Hg ions in these regions. Differences in the strain magnitudes (∊) become notable at 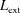 ≤ 0.5 µm: at 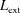 ≃ 0.3 µm the strain values in the surface layers are ∊ ≃ 10^−3^ in region III and ∊ ≃ 5 × 10^−4^ in region I.

In region II, at layer thickness 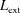 ≤ 1 µm (Fig. 3 ▸) the diffracted radiation is detected as background. This indicates a strong disorder of the structure in this region of the surface layer. Estimates show that the layer thickness is of the order of 0.2 µm.

The contribution of more components of the strain tensor (due to structural and mechanical defects) and strain relaxation near the surface is determinative in the diffraction pattern formation if the X-ray penetration depth is reduced (Lerche *et al.*, 1991 ▸). This can be used to analyze the set of X-ray topograms from one or equivalent reflections in order to improve the efficiency of X-ray topography in quantifying the inhomogeneities in the near-surface layers of crystals.

### 4.2. Ion irradiation of the CdTe surface   

The next research objects were single crystals of CdTe(111), grown by the Bridgman method, with a dislocation density of ∼5 × 10^4^ cm^−2^. Ion implantation was carried out by As ions with energy *E* = 100 keV and dose *D* = 1 × 10^15^ cm^−2^. A part of the sample surface was protected from ion influence by covering it with a mask of chemically resistant lacquer.

Under ion implantation significant disordering and damage of the surface layers are caused by the formation of excess concentrations of point defects in the two sublattices, as well as cluster formation, surface dislocations and stacking faults (Vlasov *et al.*, 2008 ▸). Implanted arsenic ions occupy anion sites of the crystal lattice. This leads to migration of ions towards the surface and, as a result, the stresses in the near-surface layers of the crystal increase.

The topogram shown in Fig. 4(*a*) ▸ was obtained in a conventional asymmetric scheme using 440 reflections. Bending and blurring of the Cu 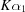 reflection indicate the presence of defects typical for such compounds in the crystal bulk (the part of region I delineated by a rectangle): structure fragmentation due to microtwinning or small-angle boundaries between crystalline grains, an inhomogeneous distribution of impurities, a deviation from the stoichiometry *etc*. For these reflections in topograms, in fact, there are no noticeable surface changes caused by implantation of As ions (region I). This is probably because the formation of the image in the topograms occurs from relatively thick layers, on which the influence of surface distortions is not significant.

At the same time it is not just mechanical damage of the sample surface that is observed in topograms (Fig. 4 ▸ *b* and 4 ▸ *c*): micro-scratches as well as the boundaries between the implanted and not-implanted regions and their different reflectivity can be seen, too. Individual local regions where the X-ray beam does not diffract are detected in the absorption and partly in the extinction contrast (hill-like inclusions 3 in Fig. 4 ▸). The sizes of these regions are from 60 to 300 µm and their density is ∼10^3^ cm^−2^. In some cases these regions can be crystalline or amorphous inclusions of another phase or pores. At 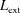 ≃ 1.65 µm (Fig. 4 ▸ *c*), in the not-implanted region, blurring and fusion of Cu 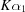 and Cu 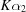 reflections are observed.

The well known software package *SRIM-2008* (http://www.srim.org/#SRIM) was used to specify the model, adequately describing the defect formation due to the implantation of As ions in CdTe. During simulation it was assumed that point defects are generated only by elastic collisions of implanted ions with lattice atoms, when the energy is transferred from the implant to the CdTe nuclear subsystem. Calculations showed that energy losses of As ions on the nuclei of lattice atoms predominate during the implantation. The average free length of the ion implant is ∼0.6 µm. The maximum concentration of implanted As ions corresponds to a depth of about 0.4 µm. Moreover, the maxima of the depth dependences of the matrix ion (Cd and Te) displacements and nuclear energy losses coincide and correspond to a depth of ∼0.2 µm. This was confirmed by the data obtained from the analysis of profiles of As distribution using the SIMS method (Vlasov *et al.*, 2008 ▸).

The strain profile was obtained by the numerical solution of the Takagi equations (Takagi, 1962 ▸), and the maximum strain value was found to be Δ*d*/*d* ≃ 2.5 × 10^−3^, which corresponds to a depth of ∼0.15 µm. The range of significant surface distortions extends to a depth of 0.3–0.35 µm, and the total thickness of the distorted layer is up to 0.9 µm

### 4.3. Implantation of CdHgTe/CdTe by As ions to different doses   

Cd_1−*x*_Hg*_x_*Te/CdTe epitaxial layers are inhomogeneous and characterized by a fragmented fine-grained surface structure (Fig. 5 ▸). They contain small-angle boundaries and single inclusions (usually Te). There is an insignificant bending of the planes. The average dislocation density is ∼5 × 10^5^ cm^−2^ (Fodchuk *et al.*, 2007 ▸).

Two stages of implantation were used to obtain a more homogeneous profile of the implanted layer, which substantially affects the electrophysical properties and structural perfection of Cd_1−*x*_Hg*_x_*Te/CdTe epitaxial layers. Such implantation is carried out by changing the type of implanted ions, their doses and their energies. In our case the first dose of As ions is *D*_1_ = 10^15^ cm^−2^ (Figs. 5 ▸ *b* and 5 ▸ *c*, region II), and then part of the crystal is irradiated with an additional dose *D*_2_ = 5 × 10^14^ cm^−2^ (Figs. 5 ▸ *b* and 5 ▸ *c*, region III). The energy of the implanted ions is 100 keV.

The reflectivity of the surface layers decreases when the dose of As ion implantation increases in the different parts of the sample owing to significant structural disorder and damage of the surface layers. The thickness of such layers can be determined from a complex analysis of the rocking curve characteristics and topogram features (Fig. 5 ▸): ∼0.29 µm for the first stage of implantation (*D*_1_) and 0.38 µm for the second stage (*D*_1_ + *D*_2_). The thickness of the layer that corresponds to structural disorder after both stages of ion implantation is ∼1.5 µm.

As in previous cases, the intensity of the reflected radiation originates from the relatively thin near-surface layers, so here distortions and local strains from defects significantly affect the formation of the kinematic image of defects on topograms.

## 5. Conclusions   

The skew-asymmetric Bragg diffraction scheme gives new possibilities for studying the morphology of the surface of semiconductor materials and structural changes after various surface processing scenarios.

With a decrease of the X-ray penetration depth, the contribution to the diffraction pattern of a larger number of components of the elastic distortion tensor and the relaxation field of deformations from structural and mechanical defects increases. This increases the sensitivity and efficiency of X-ray topography and rocking curves in the quantitative characterization of inhomogeneities in the near-surface layers of crystals.

The use of the technical and logistic capabilities of modern synchrotron topography and diffractometry in a skew-asymmetric scheme would allow *in situ* selective investigation of structural changes with high resolution and their quantitative values in thin near-surface layers of crystalline materials from a series of topograms and rocking curves under different influences.

## Figures and Tables

**Table 1:** The wavelengths and diffracted planes (*hkl*) for a skew-asymmetric diffraction geometry for Si, Ge, CdTe and InSb single crystals

No.	Crystal	Surface	*hkl*	λ (Å)	θ (°)	θ − ψ (°)	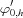 (°)	*L*_ext_ (µm at φ = 90°)	φ (°) (*L*_ext_ = 0.1 µm)
1	Si	(100)	331	1.7889, Co *K*α_1_	45.88	−0.62	11.93	13.11	11.95
		(111)	311	1.5405, Cu *K*α_1_	28.06	−1.41	19.55	10.69	19.56

2	Ge	(111)	220	2.2896, Cr *K*α_1_	34.91	−0.35	8.5	2.34	8.85
		(111)	113	1.6578, Ni *K*α_1_	29.07	−0.42	10.08	4.42	10.2

3	GaAs	(111)	311	1.6578, Ni *K*α_1_	29.10	−0.50	10.31	2.91	10.52

4	InSb	(111)	511	1.5405, Cu *K*α_1_	38.20	−0.74	13.59	6.21	13.63

5	CdTe	(111)	511	1.5405, Cu *K*α_1_	38.14	−0.80	13.75	6.74	13.78

6	CdHgTe	(111)	511	1.5405, Cu *K*α_1_	38.27	−0.67	14.32	5.82	14.36
